# Особенности бессимптомной феохромоцитомы/параганглиомы по данным клинико-лабораторного обследования и компьютерной томографии

**DOI:** 10.14341/probl13639

**Published:** 2026-09-08

**Authors:** Д. В. Реброва, С. Н. Фогт, Р. А. Черников, И. В. Слепцов, Е. А. Федоров, И. К. Чинчук, Ш. Ш. Шихмагомедов, Н. В. Ворохобина, Е. А. Згода, А. А. Семенов, В. Ф. Русаков, Л. М. Краснов

**Affiliations:** Санкт-Петербургский государственный университет, Клиника высоких медицинских технологий им. Н.И. Пирогова Россия; Saint Petersburg State University, Saint Petersburg State University Hospital Russian Federation; Северо-Западный государственный медицинский университет им. И.И. Мечникова Россия; North-Western State Medical University named after I.I. Mechnikov Russian Federation

**Keywords:** феохромоцитома, параганглиома, компьютерная томография, инциденталома надпочечника, адреналэктомия, pheochromocytoma, paraganglioma, tomography, X-ray computed, adrenal incidentaloma, adrenalectomy

## Abstract

**ОБОСНОВАНИЕ:**

ОБОСНОВАНИЕ. Несмотря на то, что в классическом варианте феохромоцитомы и параганглиомы (ФХЦ/ПГ) характеризуются яркими клиническими признаками, существует категория пациентов, у которых это заболевание протекает бессимптомно. Несмотря на это, и при отсутствии клинических проявлений у больных с ФХЦ/ПГ сохраняется риск развития жизнеугрожающего гипертонического криза и других сердечно-сосудистых осложнений. Клинико-лабораторные и лучевые характеристики бессимптомной формы заболевания изучены недостаточно и представляют собой большой научный и практический интерес.

**ЦЕЛЬ:**

ЦЕЛЬ. Проанализировать клинико-лабораторные и лучевые характеристики бессимптомных ФХЦ/ПГ в сравнении с симптоматическими.

**МАТЕРИАЛЫ И МЕТОДЫ:**

МАТЕРИАЛЫ И МЕТОДЫ. В анализ включено 347 пациентов, прооперированных в Клинике высоких медицинских технологий им. Н.И. Пирогова Санкт-Петербургского государственного университета, с гистологически подтвержденной ФХЦ/ПГ. Ретроспективно проанализированы данные по демографическим характеристикам, анамнезу основного и сопутствующих заболеваний, проведена оценка результатов лабораторных исследований, а также компьютерной томографии с контрастированием.

**РЕЗУЛЬТАТЫ:**

РЕЗУЛЬТАТЫ. Выборка пациентов с ФХЦ/ПГ включала 45 случаев без характерных клинических признаков гиперсекреции катехоламинов. В подгруппе бессимптомных больных в сравнении с остальными обнаружен более молодой возраст (43 [33; 51] против 48 [37; 57] лет), меньший максимальный размер опухоли (34 [27; 53] против 45 [34; 60] мм), более низкое соотношение метанефрин/норметанефрин (0,29 [0,10; 0,79] против 0,51 [0,09; 1,23]).

По данным компьютерной томографии в группе бессимптомной ФХЦ/ПГ пациенты имели несколько более высокие показатели рентгенологической плотности опухоли в артериальную, венозную и отсроченную фазы в сравнении с больными с симптоматическим заболеванием, однако абсолютный и относительный проценты вымывания контраста не различались.

**ЗАКЛЮЧЕНИЕ:**

ЗАКЛЮЧЕНИЕ. Более молодой возраст и меньший максимальный размер опухоли у пациентов с бессимптомными ФХЦ/ПГ в сравнении с симптоматическими свидетельствуют о том, что первый тип опухолей выявляется на более ранней стадии по сравнению со вторыми.

Более низкое усредненное соотношение метанефрин/норметанефрин может указывать на большую распространенность норадреналинергического типа секреции катехоламинов при бессимптомной форме ФХЦ/ПГ.

Выраженных различий по данным визуализирующих методов диагностики между бессимптомными и симптоматическими опухолями, не обнаружено.

## Abstract (en)

**BACKGROUND:**

BACKGROUND: Although pheochromocytomas and paragangliomas (PPGLs) classically present with distinct clinical signs, there is a category of patients with asymptomatic disease course. Despite the absence of clinical manifestations, these patients retain the risk of life-threatening hypertensive crisis and other cardiovascular complications. The clinical, laboratory, and imaging characteristics of asymptomatic PPGLs remain insufficiently studied and are of significant scientific and practical interest.

**AIM:**

AIM: To analyze the clinical, laboratory, and imaging characteristics of asymptomatic PPGLs compared to symptomatic ones.

**MATERIALS AND METHODS:**

MATERIALS AND METHODS: The analysis included 347 patients with histologically confirmed PPGL who underwent surgery at the Saint Petersburg State University Hospital. Retrospective analysis encompassed demographic data, medical history (primary and comorbid conditions), laboratory findings, and computed tomography (CT) data. RESULTS: The PPGLs patient cohort included 45 cases without clinical signs of catecholamine hypersecretion. The asymptomatic subgroup, compared to symptomatic patients, exhibited younger age (43 [33; 51] vs. 48 [37; 57] years); smaller maximum tumor size (34 [27; 53] vs. 45 [34; 60] mm); lower metanephrine/normetanephrine ratio (0.29 [0.10; 0.79] vs. 0.51 [0.09; 1.23]).

CT imaging revealed slightly higher radiodensity values in asymptomatic PPGLs during arterial, venous, and delayed phases compared to symptomatic tumors. However, absolute and relative washout percentages did not differ significantly.

**CONCLUSION:**

CONCLUSION: The younger age and smaller tumor size in asymptomatic PPGL patients suggest earlier detection of the disease compared to symptomatic cases. The lower metanephrine/normetanephrine ratio may indicate a higher prevalence of the norepinephrine-secreting phenotype in asymptomatic PPGLs. No pronounced differences were found in imaging characteristics between asymptomatic and symptomatic tumors.

## ОБОСНОВАНИЕ

Феохромоцитомы и параганглиомы (ФХЦ/ПГ) представляют собой нейроэндокринные опухоли, происходящие из хромаффинных клеток мозгового вещества надпочечников или вненадпочечниковой параганглионарной ткани [1–3]. Клинические проявления ФХЦ/ПГ определяются способностью этих опухолей синтезировать и высвобождать катехоламины, воздействующие на адренергические рецепторы в различных тканях и органах [1]. Клиническая картина при ФХЦ/ПГ крайне вариабельна и включает множество симптомов, однако у большинства пациентов выявляются типичные клинические признаки, включающие головную боль, сердцебиение, чувство тревоги и профузное потоотделение [1]. В классическом варианте диагностический поиск обычно инициируется при выявлении у пациента высокой артериальной гипертензии, после чего в результате обследования выявляется катехоламин-продуцирующая опухоль. Однако так происходит далеко не всегда: существует категория пациентов с ФХЦ/ПГ, у которых заболевание протекает бессимптомно («клинически молчащие» опухоли) [2][4][5]. Такие случаи преимущественно являются инциденталомами надпочечников, то есть образованиями, выявляемыми случайно при проведении визуализирующих исследований по поводу других состояний (например, абдоминальной боли, мочекаменной болезни, пневмонии) или в рамках профилактического обследования.

Несмотря на отсутствие типичной симптоматики, у пациентов с бессимптомными ФХЦ/ПГ сохраняется риск развития жизнеугрожающего гипертонического криза. Такой криз может возникнуть спонтанно или быть спровоцированным хирургическим вмешательством. Подобные случаи острой гемодинамической нестабильности описаны в ряде исследований, когда у пациента с инциденталомой надпочечника криз развивался во время операции по поводу других заболеваний брюшной полости или непосредственно при выполнении адреналэктомии для удаления самой инциденталомы [6–10]. Описаны случаи острой сердечной недостаточности, жизнеугрожающей аритмии и остановки сердца на фоне бессимптомной ФХЦ [11][12]. Таким образом, бессимптомные ФХЦ/ПГ представляют собой потенциально опасное для жизни заболевание, особенно учитывая тот факт, что без клинической симптоматики вероятность установления верного диагноза значительно ниже. Учитывая редкость ФХЦ/ПГ, клинико-лабораторные и лучевые характеристики бессимптомной формы заболевания изучены недостаточно и представляют собой большой научный и практический интерес.

## ЦЕЛЬ ИССЛЕДОВАНИЯ

Выявить особенности бессимптомной ФХЦ/ПГ по данным клинико-лабораторного обследования и компьютерной томографии (КТ) путем сравнения с симптоматическими опухолями.

## МАТЕРИАЛЫ И МЕТОДЫ

## Место и время проведения исследования

Место исследования. Клиника высоких медицинских технологий им. Н.И. Пирогова Санкт-Петербургского государственного университета (КВМТ СПбГУ).

Время исследования. С января 2011 по декабрь 2022 гг.

## Изучаемые популяции

В анализ включено 347 пациентов, прооперированных в КВМТ СПбГУ за указанный период, с гистологически подтвержденной ФХЦ/ПГ.

## Способ формирования выборки из изучаемой популяции

Выборка пациентов сплошным способом.

## Дизайн исследования

## Методы

Ретроспективно собраны данные по демографическим характеристикам и весоростовым показателям пациентов (пол, возраст, рост, масса тела), анамнезу основного заболевания, значимым сопутствующим заболеваниям. Подробно проанализированы анамнестические данные по артериальной гипертензии. Бессимптомной ФХЦ/ПГ считали гистологически подтвержденную опухоль при отсутствии выявленных симптомов гиперсекреции катехоламинов.

Проведена оценка данных лабораторных исследований по результатам анализов плазмы и/или мочи на общие нефракционированные метанефрины, а также на фракционированные общие и свободные метанефрин и норметанефрин [13].

Всем пациентам была проведена визуализация надпочечников при помощи КТ. В анализ включены только исследования, выполненные в КВМТ СПбГУ. Собранные данные включали максимальный размер новообразования, его локализацию, рентгенологическую плотность нативно, а также после введения 100 мл контрастного вещества, содержащего 370 мг йода, в артериальную фазу, венозную фазу и отсроченную фазу контрастирования (через 10 минут). Абсолютный и относительный проценты вымывания контраста (АПВК и ОПВК) рассчитывали по формулам:

;

;

где А — плотность новообразования в артериальную фазу после введения контраста,

B — плотность новообразования через 10 минут после введения контраста,

С — нативная плотность новообразования.

## Статистический анализ

Статистическая обработка осуществлялась с использованием программного пакета Statistica v. 10.0. Количественные данные представлены в виде Me [ LQ; HQ], где Me — медиана, LQ — нижний квартиль, HQ — верхний квартиль.

Сравнение количественных переменных осуществлялось с помощью критерия Манна-Уитни. Сравнение категориальных переменных проводилось с использованием критерия хи-квадрат с поправкой Йетса либо двустороннего точного критерия Фишера при числе наблюдений в ячейках таблицы сопряженности менее пяти.

## Этическая экспертиза

Исследование рассмотрено на заседании Комитета по биомедицинской этике КВМТ СПбГУ, протокол №08/25 от 17.07.2025. Учитывая ретроспективный характер исследования и отсутствие персональных идентификационных данных, неинтервенционный дизайн исследования, письменного согласия пациентов и специального одобрения этическим комитетом не требуется.

## РЕЗУЛЬТАТЫ

В анализ включены данные 347 пациентов с ФХЦ/ПГ, из которых 45 имели бессимптомное заболевание, а 302 — симптоматическое (163 — с пароксизмальной формой, 15 — с постоянной, 124 — со смешанной).

Демографическая характеристика пациентов с ФХЦ/ПГ с симптоматическим и бессимптомным течением заболевания представлена в таблице 1.

**Table:** Таблица 1. Демографическая характеристика пациентов с феохромоцитомой/параганглиомой Примечание: * — критерий хи-квадрат с поправкой Йетса, ** — критерий Манна-Уитни.

Показатель	Пациенты с бессимптомной ФХЦ/ПГ(n=45)	Пациенты с симптоматической ФХЦ/ПГ(n=302)	Значение p
Пол мужской	13 (28,9%)	93 (30,8%)	0,920*
Возраст при госпитализации, годы	43 [ 33; 51]	48 [ 37; 57]	0,040**
Рост, см	168 [ 159; 173]	166 [ 161; 172]	0,743**
Масса тела, кг	63 [ 58; 74]	70 [ 60; 84]	0,076**
Индекс массы тела, кг/м²	23,1 [ 21,0; 25,8]	24,8 [ 22,2; 28,7]	0,033**

Обследованные пациенты были сопоставимы по половому составу. Возраст на момент госпитализации по поводу ФХЦ/ПГ был меньше у пациентов с бессимптомной опухолью в сравнении с симптоматическим заболеванием. В первой группе пациентов также отмечены несколько более низкие масса тела и индекс массы тела (ИМТ).

У девяти пациентов опухоль была представлена параганглиомой вненадпочечниковой локализации, из них у семи имелись характерные симптомы заболевания, у двух они выявлены не были. При сравнении частот асимптоматического течения заболевания у пациентов с ФХЦ/ПГ не было выявлено значимых различий (p=0,611, точный двусторонний критерий Фишера).

В таблице 2 и на рисунке 1 представлены показатели артериального давления (АД) у пациентов с ФХЦ/ПГ. У пациентов с бессимптомной ФХЦ/ПГ закономерно отмечались значительно более низкие значения максимальных систолического и диастолического артериального давления в сравнении с пациентами с симптоматическим заболеванием, в то же время цифры рабочего АД были тоже несколько ниже.

**Table:** Таблица 2. Показатели артериального давления у пациентов с феохромоцитомой/параганглиомой Примечание: * — критерий Манна-Уитни.

Показатель	Пациенты с бессимптомной ФХЦ/ПГ(n=45)	Пациенты с симптоматической ФХЦ/ПГ(n=302)	Значение p
Максимальное систолическое АД, мм рт.ст.	135 [ 120; 147]	200 [ 170; 220]	<0,001*
Максимальное диастолическое АД, мм рт.ст.	80 [ 80; 90]	110 [ 100; 120]	<0,001*
Рабочее систолическое АД, мм рт.ст.	120 [ 110; 120]	120 [ 120; 130]	0,007*
Рабочее диастолическое АД, мм рт.ст.	80 [ 70; 80]	80 [ 70; 85]	0,061*

**Figure:**
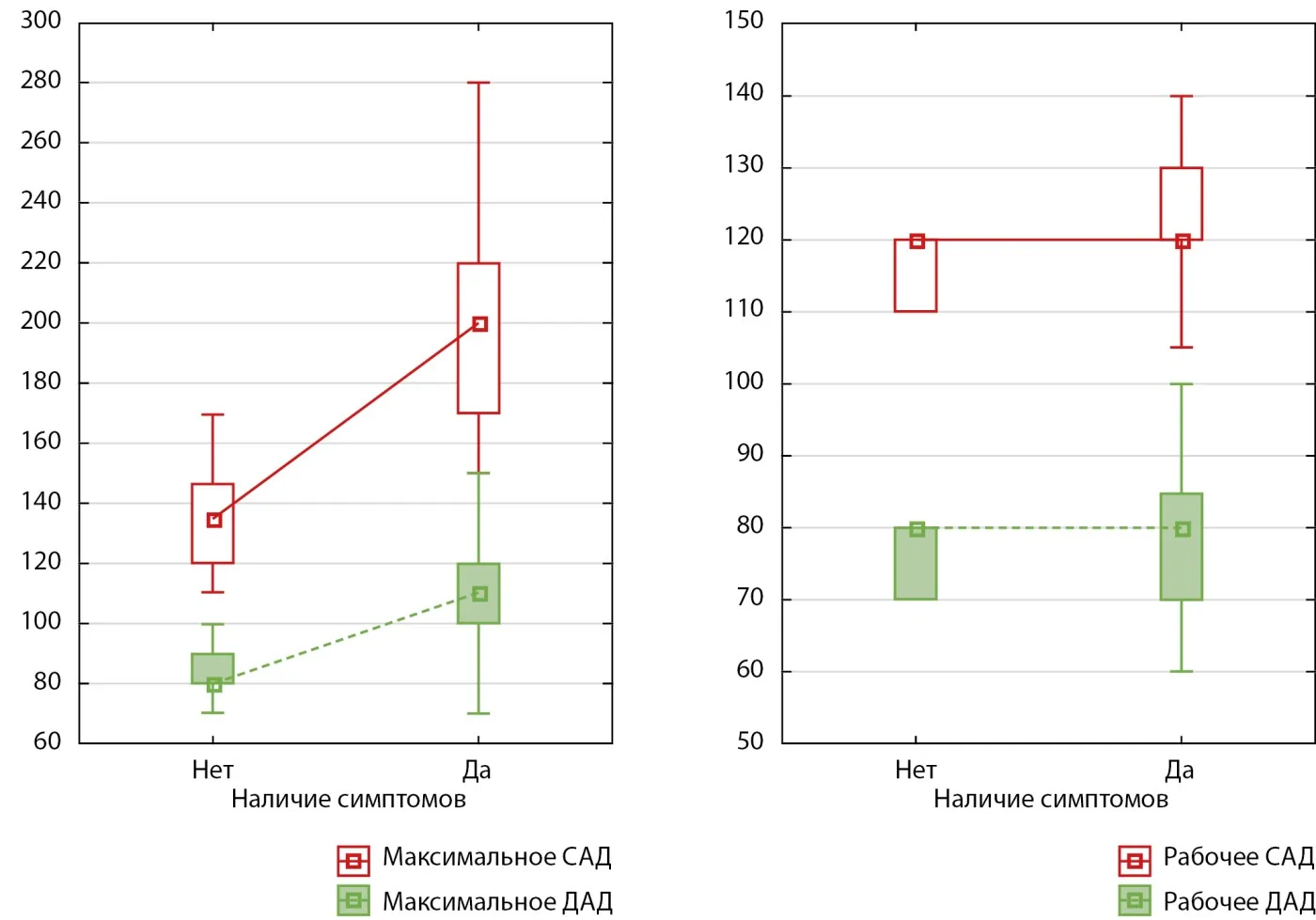
Рисунок 1. Максимальное и рабочее систолическое и диастолическое артериальное давление у пациентов с феохромоцитомой/параганглиомой. Примечание: квадрат в прямоугольнике — медиана, границы прямоугольника — верхний и нижний квартили, усы — диапазон без выбросов.

У большинства пациентов КТ надпочечников была выполнена в КВМТ СПбГУ, в связи с чем доступна к пересмотру и ретроспективному анализу. Основные показатели приведены в таблице 3.

**Table:** Таблица 3. Показатели компьютерной томографии надпочечников у пациентов с феохромоцитомой/параганглиомой Примечание: * — критерий хи-квадрат с поправкой Йетса, ** — критерий Манна-Уитни.

Показатель	Пациенты с бессимптомной ФХЦ/ПГ(n=45)	Пациенты с симптоматической ФХЦ/ПГ(n=302)	Значение p
Локализация опухоли: право-/лево-/двусторонняя	27/10/2	124/115/27	0,031*
Однородность структуры опухоли: однородная/неоднородная	26/38 (68,4%)	164/264 (62,1%)	0,566*
Максимальный размер опухоли, мм	34 [ 27; 53]	45 [ 34; 60]	0,008**
Нативная рентгенологическая плотность опухоли, HU	35 [ 25; 40]	30 [ 25; 37]	0,150**
Рентгенологическая плотность опухоли в артериальную фазу, HU	100 [ 75; 136]	89 [ 65; 116]	0,040**
Рентгенологическая плотность опухоли в венозную фазу, HU	105 [ 80; 124]	90 [ 75; 107]	0,013**
Рентгенологическая плотность опухоли в отсроченную фазу, HU	67 [ 57; 71]	60 [ 52; 70]	0,038**
Абсолютный процент вымывания контраста, %	52,9 [ 22,1; 72,8]	53,3 [ 15,8; 67,8]	0,624**
Относительный процент вымывания контраста, %	36,2 [ 12,5; 53,5]	34,1% [ 8,3; 45,8]	0,454**

Отмечено, что в группе бессимптомной ФХЦ/ПГ реже встречалась левосторонняя локализация опухоли, пациенты имели меньший размер неоплазии и более высокие показатели рентгенологической плотности новообразования в артериальную, венозную и отсроченные фазы в сравнении с больными с симптомным течением заболевания. Абсолютный процент вымывания контраста (АПВК) и относительный процент вымывания контраста (ОПВК) (рис. 2), частота однородной структуры опухоли по сравниваемым группам не различались.

**Figure:**
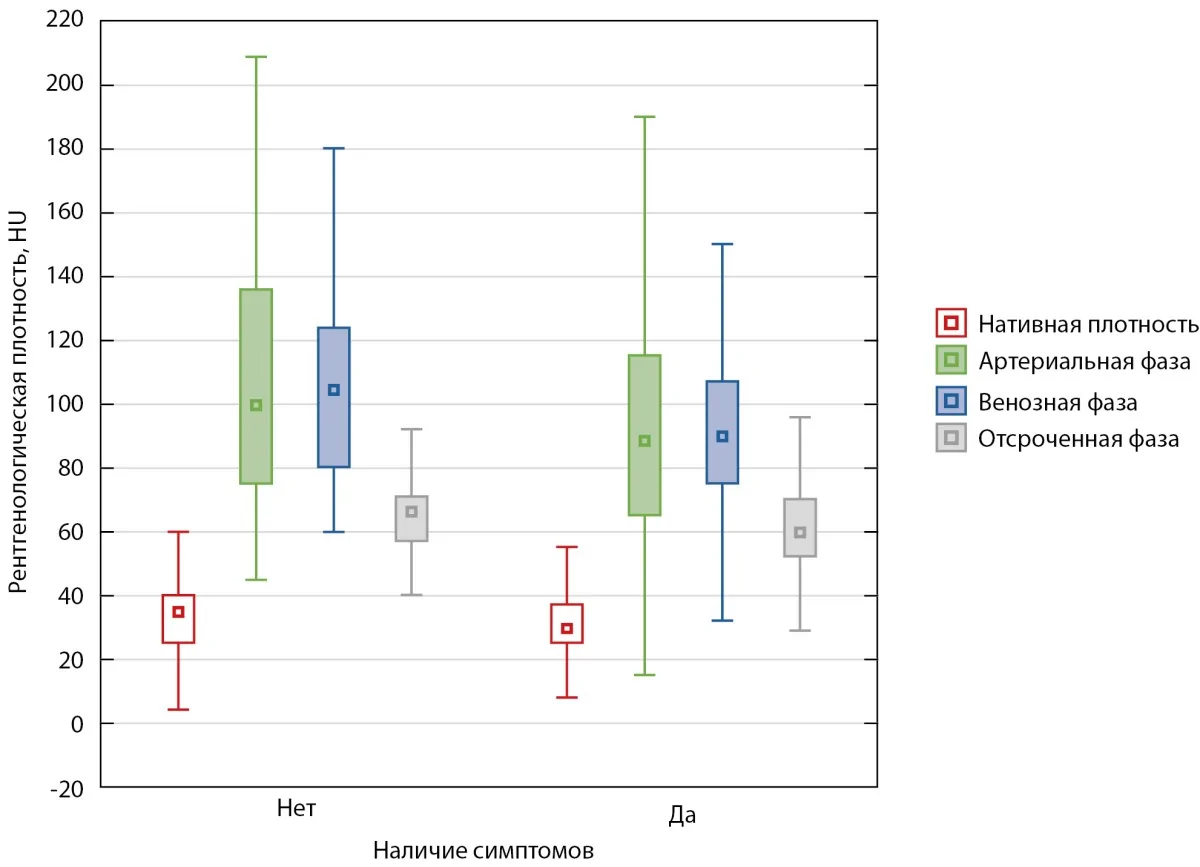
Рисунок 2. Нативная рентгенологическая плотность опухоли и ее динамика после введения контраста у пациентов с феохромоцитомой/параганглиомой. Примечание: квадрат в прямоугольнике — медиана, границы прямоугольника — верхний и нижний квартили, усы — диапазон без выбросов.

Для исследовательской цели проанализированы показатели соотношения метанефрина и норметанефрина у пациентов, так как известно, что профиль опухолевой секреции влияет на клиническую картину заболевания. Поскольку определение метанефрина и норметанефрина пациентов проводилось множеством различных методов (в плазме или в моче, свободные или общие фракции), выборка бессимптомных ФХЦ/ПГ не позволила провести анализ в соответствии с каждым отдельным методом определения метанефринов. Тем не менее при допущении, что соотношение метанефрин/норметанефрин не зависит значимо от метода определения метанефринов, было возможно рассчитать общее соотношение метанефрин/норметанефрин для 30 пациентов с бессимптомной опухолью и для 186 с симптоматической. Для пациентов, у которых были доступны результаты определения метанефринов несколькими методами, использовано усредненное значение показателя.

Соотношение метанефрин/норметанефрин составило в группах бессимптомной и симптоматической ФХЦ/ПГ 0,29 [ 0,10; 0,79] и 0,51 [ 0,09; 1,23] соответственно. Несмотря на то, что показатели статистически значимо не различались (p=0,359, критерий Манна-Уитни), более высокие значения были отмечены во второй группе пациентов. В группе бессимптомной ФХЦ/ПГ максимальное значение соотношения метанефрин/норметанефрин составило 2,03, тогда как 32/186 (17,2%) человек с симптоматическими опухолями имели значение выше этого уровня (p=0,022, точный критерий Фишера).

Учитывая специфический тип секреторной активности у параганглиом, дополнительно проанализированы соответствующие показатели только у пациентов с ФХЦ. Соотношение метанефрин/норметанефрин составило в группах бессимптомной и симптоматической ФХЦ/ПГ 0,29 [ 0,10; 0,83] и 0,52 [ 0,10; 1,23] (p=0,393, критерий Манна-Уитни). В группе бессимптомной ФХЦ максимальное значение показателя составило 2,03, 28/182 (15,4%) человек с симптоматическими опухолями имели значение выше этого уровня (p=0,031, точный критерий Фишера). Таким образом, можно констатировать, что различия по показателю соотношения метанефрин/норметанефрин действительны и для пациентов с ФХЦ без включения в анализ ПГ.

Изображения сканов четырехфазной КТ ФХЦ с различными типами секреции представлены на рисунках 3–5.

**Figure:**
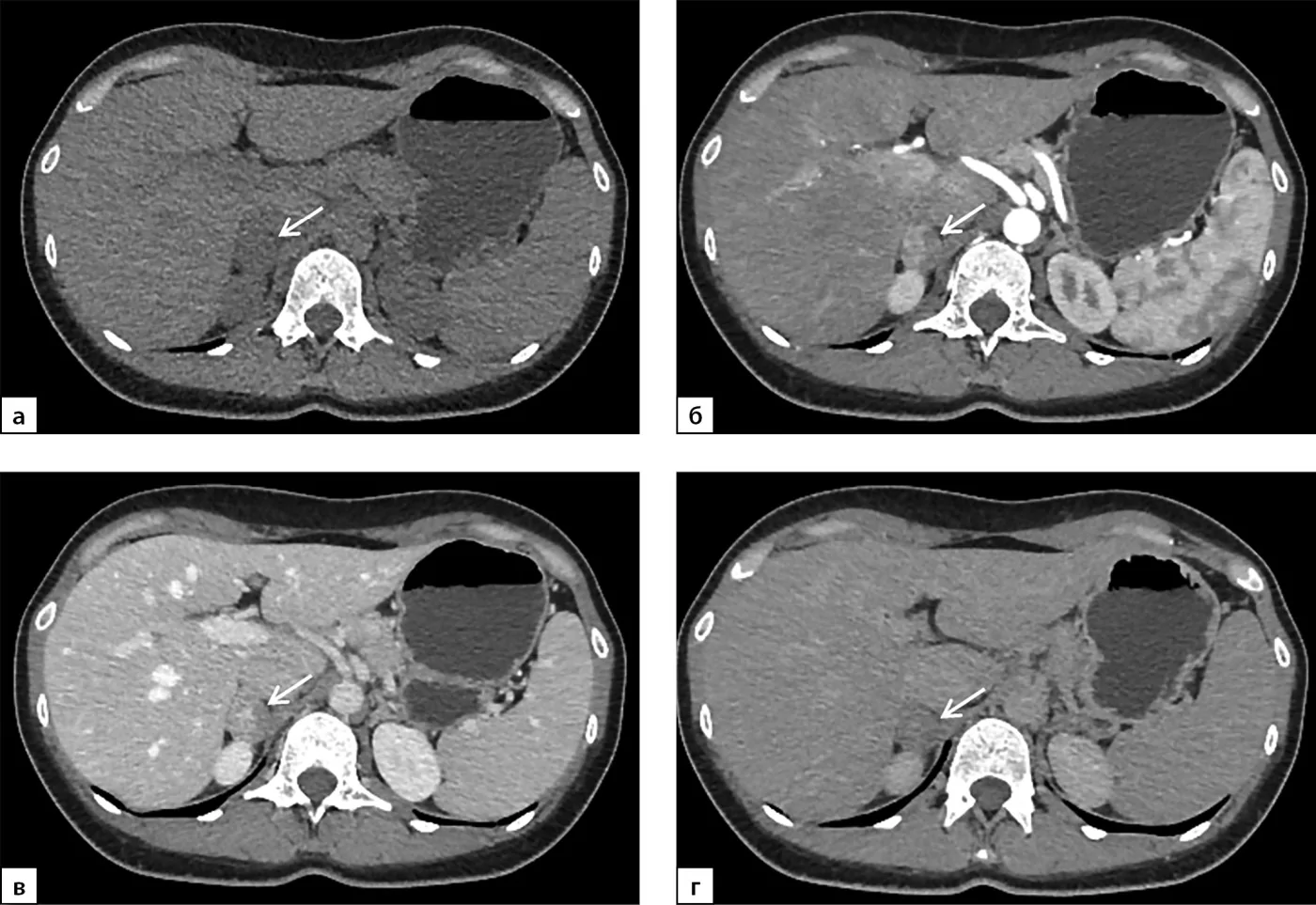
Рисунок 3. Феохромоцитома правого надпочечника размерами 26х20х26 мм, преимущественно метанефриновый тип секреции, бессимптомное течение. Компьютерная томография с контрастированием: а — нативная фаза, плотность 20 HU; б — артериальная фаза, плотность до 105 HU; в — венозная фаза, плотность до 145 HU; г — отсроченная фаза, плотность 50 HU.

**Figure:**
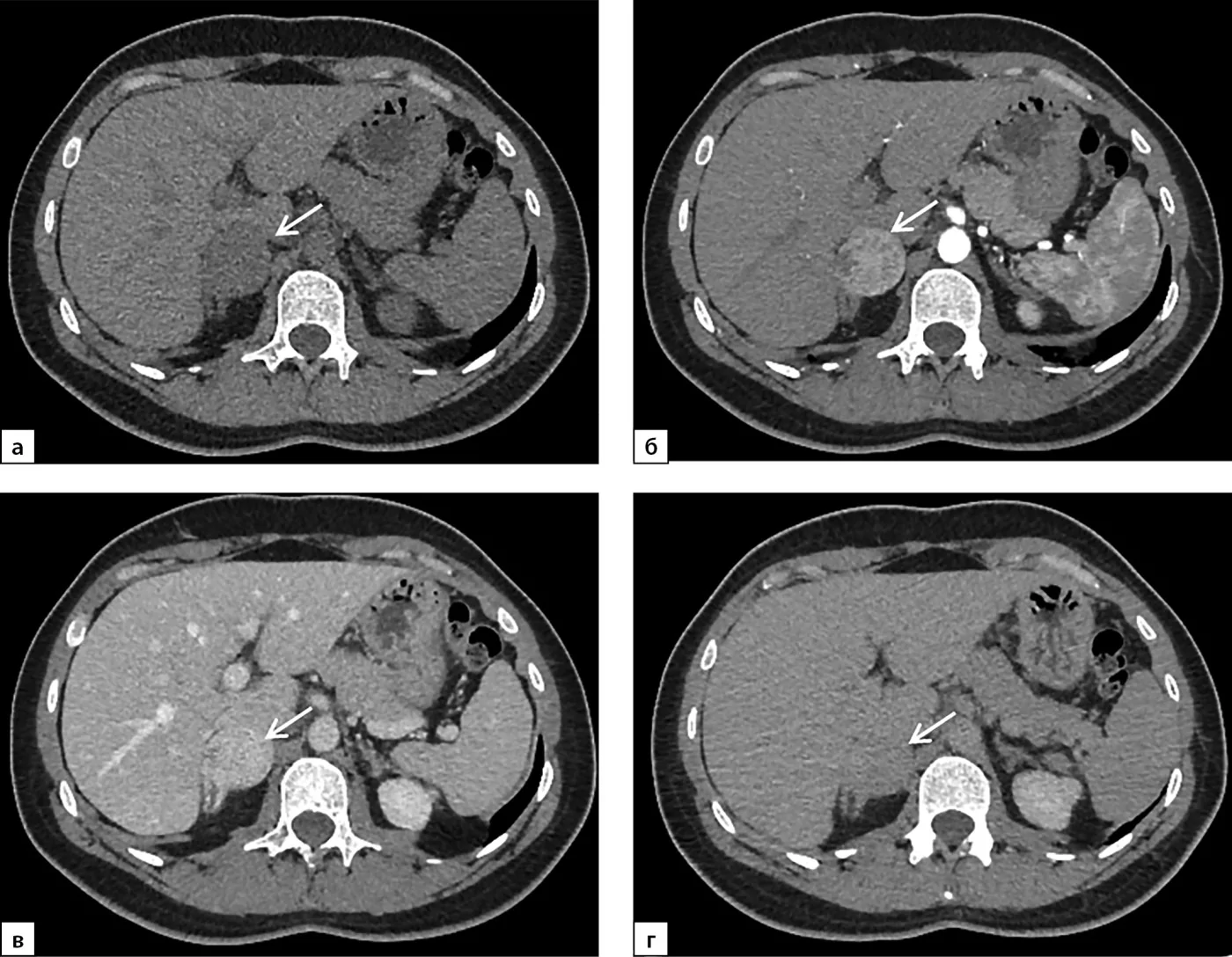
Рисунок 4. Феохромоцитома правого надпочечника размерами 45х38х32 мм, норметанефриновый тип секреции, бессимптомное течение. Компьютерная томография с контрастированием: а — нативная фаза, плотность до 35 HU; б — артериальная фаза, плотность до 106 HU; в — венозная фаза, плотность до 130 HU; г — отсроченная фаза, плотность до 82 HU.

**Figure:**
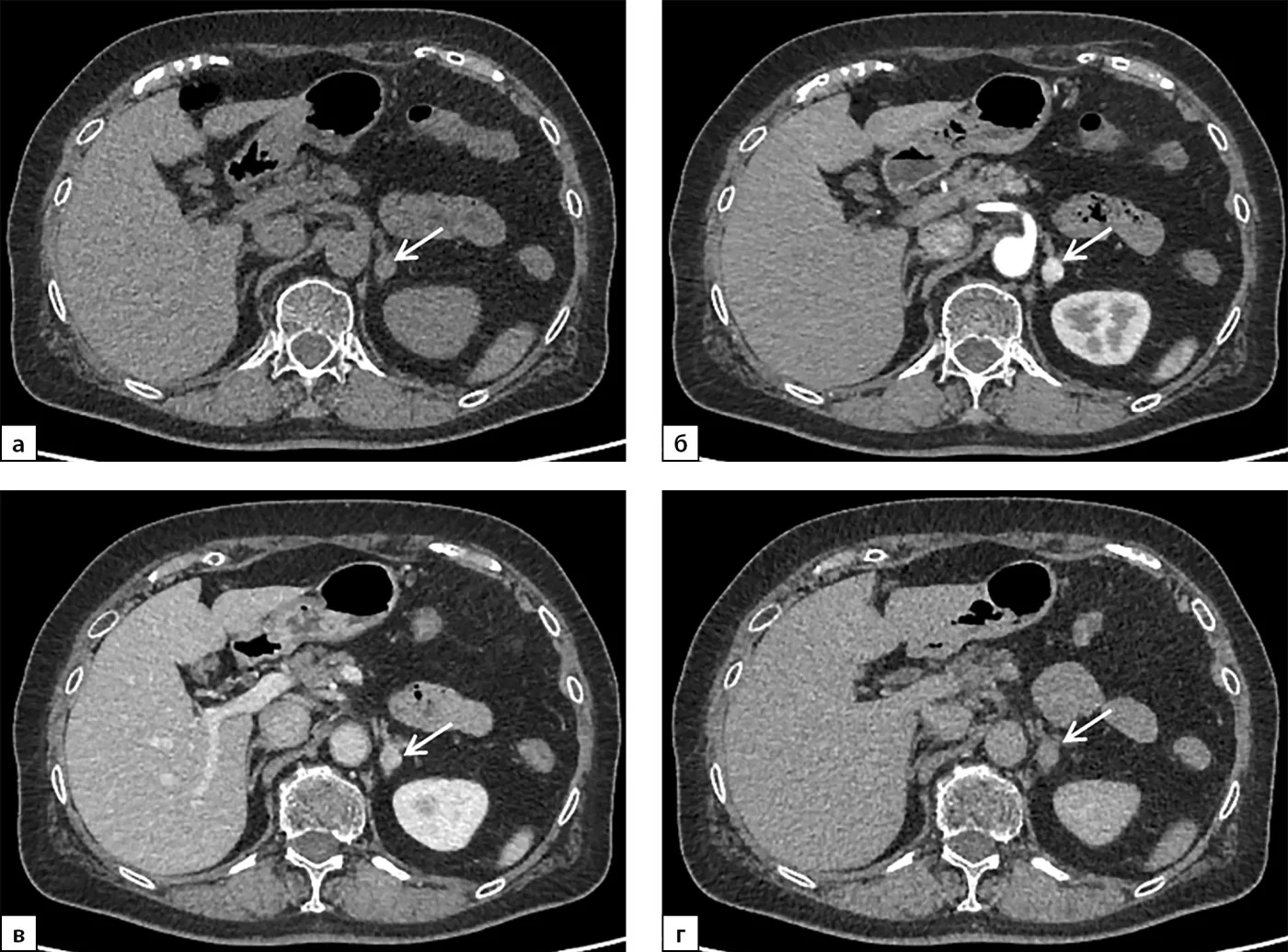
Рисунок 5. Феохромоцитома левого надпочечника размерами 11х10х11 мм, неоднородной структуры, клинически и биохимически «немая». Компьютерная томография с контрастированием: а — нативная фаза, плотность от 34 до 43 HU; б — артериальная фаза, плотность от 158 до 209 HU; в — венозная фаза, плотность от 111 до 139 HU; г — отсроченная фаза, плотность до 70 HU.

## ОБСУЖДЕНИЕ

Нами проведен анализ крупной выборки пациентов с ФХЦ/ПГ, включая бессимптомную форму заболевания. Стоит отметить, что литературные сведения по этой проблеме весьма скудные, что не дает возможность четко сопоставить результаты проведенного анализа с данными других авторов.

Согласно нашим данным, асимптоматическая ФХЦ/ПГ встречалась в 45 из 347 случаев (13,0%). При сравнении с выборкой из 230 больных в работе Тарбаевой Н.В. и соавт. бессимптомная форма встречалась существенно реже — только в 10 случаях (4,3%) [14].

При анализе демографических данных обращает на себя внимание более молодой возраст пациентов с бессимптомной ФХЦ/ПГ в сравнении с больными с симптоматическим заболеванием. У этих же пациентов отмечены меньшие значения массы тела и ИМТ. Последнее, вероятно, связано с более молодым возрастом пациентов, так как известно, что распространенность ожирения увеличивается с возрастом [15].

Характер клинического течения заболевания, согласно полученным данным, оказался не связан с видом опухоли (надпочечниковая или вненадпочечниковая ФХЦ/ПГ). Тем не менее необходимо отметить, что такой вывод не может быть сделан достоверно, учитывая ограниченное число случаев параганглиом в анализе, а также наличие сведений в литературе о секреции параганглиомами преимущественно только норадреналина [16]. Также необходимо отметить: параганглиомы головы и шеи, которые имеют парасимпатическую природу и, как следствие, значительно отличаются по симптоматике от симпатических, в нашей выборке не встречались [2].

При анализе значений АД у пациентов с бессимптомной ФХЦ/ПГ отмечались значительно более низкие значения максимальных систолического и диастолического АД в сравнении с пациентами с симптоматическим заболеванием. Данная находка закономерна, учитывая, что основным проявлением ФХЦ/ПГ является именно повышение АД. Однако стоит отметить, что в группе больных с симптоматической формой опухоли встречались также нормотензивные пациенты, клиническая картина заболевания у которых проявлялась в виде пароксизмальных приступов тахикардии, ощущений нарушений ритма сердца, головных болях, головокружениях, тошноте, болях в животе или боку и т.д. Уровни рабочего АД оказались тоже несколько ниже у пациентов с бессимптомной опухолью, что может быть связано с более молодым возрастом пациентов (ниже распространенность гипертонической болезни), а также постоянной (фоновой) секрецией катехоламинов у пациентов с симптоматической ФХЦ/ПГ.

Согласно данным КТ, в исследуемой выборке несколько чаще встречалась правосторонняя локализация опухоли. Для пациентов с бессимптомным заболеванием отмечено более выраженное преобладание правосторонней локализации опухоли, чем для симптоматических опухолей. Поскольку в литературе отсутствуют сведения о значимом превалировании частоты поражения правого надпочечника [17][18], а также ввиду отсутствия научного объяснения этому факту, вероятно, данная находка носит случайный характер и связана с относительно малым числом наблюдений.

Примечательно, что максимальный размер опухоли в случае бессимптомного течения оказался значимо меньше, чем при симптоматическом течении (медианный диаметр был меньше на 11 мм (24,4%), что примерно соответствует двукратному уменьшению объема опухоли). Следует отметить, что существует работа Kim и соавт., в которой описано 8 случаев бессимптомной ФХЦ/ПГ. Все 8 опухолей имели размер более 4 см (средний размер 45 мм) [4]. Результаты настоящего исследования несколько контрастируют с этими данными, так как медиана размера опухоли в нашей выборке составила 34 мм. В то же время необходимо отметить, что на результаты исследования Kim могли повлиять крайне малая выборка и этнический фактор (исследование проведено в Корее), а также, возможно, особенности систем здравоохранения, например, частота выполнения визуализирующих исследований органов брюшной полости у населения.

Согласно результатам исследования, в группе бессимптомной ФХЦ/ПГ пациенты имели более высокие показатели рентгенологической плотности опухоли в артериальную, венозную и отсроченную фазы в сравнении с больными с симптоматическим заболеванием, однако АПВК и ОПВК не различались. В сравнении с исследованием Kim и соавт. [4] в нашей выборке нативная плотность была выше (медиана 35 против среднего 25 HU), существенно выше оказались также АПВК (медиана 52,9 против среднего 29,0) и ОПВК (медиана 36,2 против среднего 17,7). Возможные причины расхождений для данных показателей указаны выше.

В литературных данных имеются указания о том, что наличие кровоизлияний и некрозов внутри опухоли может способствовать снижению продукции катехоламинов даже при больших размерах новообразований [2], а также о том, что момент образования некроза или кровоизлияния может сопровождаться гипертоническими кризами [9][10], т.е. симптоматические и бессимптомные опухоли могут различаться по показателю однородности структуры новообразования по данным КТ. Тем не менее, по результатам нашего исследования, доля опухолей с однородной структурой по данным КТ была примерно одинаковой у сравниваемых групп пациентов.

Анализ соотношения метанефрин/норметанефрин имеет свои ограничения, так как у разных пациентов он выполнялся разными методами. Тем не менее полученные данные могут указывать на более низкий показатель в случае бессимптомной ФХЦ/ПГ. Это может говорить о том, что преимущественно норадреналинергический тип секреции катехоламинов может быть более распространен при бессимптомной ФХЦ/ПГ. Дополнительный проведенный анализ только на феохромоцитомах имел аналогичные результаты, что и с общей выборкой, а это всвою очередь показывает: включение в анализ параганглиом (которые обладают норадреналиновым типом секреции) не является причиной выявленных закономерностей в отношении соотношения метанефрин/норметанефрин.

Выявленные в нашей работе закономерности позволяют выдвинуть предположение, что бессимптомные ФХЦ/ПГ зачастую представляют собой опухоли, которые были выявлены на более ранней стадии в сравнении с симптоматическими новообразованиями. Данную гипотезу поддерживает, прежде всего, нахождение существенно меньшего размера опухоли и более молодого возраста у пациентов с бессимптомными ФХЦ/ПГ. Механизм конверсии бессимптомной опухоли в симптоматическую может быть связан с постепенным увеличением опухолевой массы (и, соответственно, секретирующих клеток) либо изменением свойств самой опухоли, связанного, например, с ростом клона клеток, секретирующих катехоламины. Также нельзя исключить, что опухоль может со временем менять паттерн секреции катехоламинов, который зависит, в первую очередь, от экспрессии фенилэтаноламин-N-метилтрансферазы, белка, конвертирующего норадреналин в адреналин. Известно, что норадреналинергический тип секреции характеризуется более мягким или даже бессимптомным течением заболевания (за счет меньшей прессорной активности норадреналина и также постепенного снижения чувствительности к нему адренорецепторов) [2]. Полученные в нашем исследовании данные, указывающие по косвенным признакам на преобладание норадреналинергического типа секреции при бессимптомных опухолях, поддерживают данную гипотезу.

Определенную роль в конверсии ФХЦ/ПГ в симптоматическую также могут играть изменение кровоснабжения различных участков опухоли, возникновение некрозов и кровоизлияний в новообразовании, которые могут спровоцировать выброс накопленных в клетках опухоли катехоламинов [9][10].

Причины выявленных при КТ различий между бессимптомными и симптоматическими ФХЦ/ПГ (повышение рентгенологической плотности в артериальную, венозную и отсроченную фазы) не вполне ясны. Возможно, они являются случайными и обусловлены относительно небольшим числом наблюдений, в то же время нельзя исключить, что рентгенологическая плотность опухоли с ростом меняется вследствие изменения структуры, появления сначала мелких, а потом и крупных кровоизлияний и некрозов. Выраженных рентгенологических различий между бессимптомными и симптоматическими опухолями, которые можно было бы использовать в диагностических целях, выявлено не было.

## ЗАКЛЮЧЕНИЕ

Проведен анализ крупной выборки из 347 пациентов с ФХЦ/ПГ, включая 45 случаев без клинических признаков гиперсекреции катехоламинов. Более молодой возраст и меньший максимальный размер опухоли у пациентов с бессимптомными ФХЦ/ПГ в сравнении с симптоматическими свидетельствуют о том, что первый тип опухолей выявляется на более ранней стадии по сравнению со вторыми.

Более низкое усредненное соотношение метанефрин/норметанефрин может указывать на большую распространенность норадреналинергического типа секреции катехоламинов при бессимптомной форме ФХЦ/ПГ.

Выраженных различий, по данным визуализирующих методов диагностики, между бессимптомными и симптоматическими опухолями не обнаружено.

## ДОПОЛНИТЕЛЬНАЯ ИНФОРМАЦИЯ

Источники финансирования. Работа выполнена по инициативе авторов без привлечения финансирования.

Конфликт интересов. Авторы декларируют отсутствие явных и потенциальных конфликтов интересов, связанных с содержанием настоящей статьи.

Участие авторов. Все авторы одобрили финальную версию статьи перед публикацией, выразили согласие нести ответственность за все аспекты работы, подразумевающую надлежащее изучение и решение вопросов, связанных с точностью или добросовестностью любой части работы.
